# Lower Bone Mineral Density in Patients with Parkinson’s Disease: A Cross-Sectional Study from Chinese Mainland

**DOI:** 10.3389/fnagi.2015.00203

**Published:** 2015-10-27

**Authors:** Huimin Gao, Xiaobo Wei, Jinchi Liao, Rui Wang, Jiehua Xu, Xu Liu, Xiaoping Pan, Ze Li, Zhong Li, Ying Xia, Qing Wang

**Affiliations:** ^1^Department of Neurology, The Third Affiliated Hospital of Sun Yat-Sen University, Guangzhou, China; ^2^Department of Nuclear Medicine, The Third Affiliated Hospital of Sun Yat-Sen University, Guangzhou, China; ^3^Department of Neurology, Guangzhou First People’s Hospital, Guangzhou, China; ^4^Department of Neurology, The Sixth Affiliated Hospital of Sun Yat-Sen University, Guangzhou, China; ^5^Department of Neurosurgery, The University of Texas Medical School at Houston, Houston, TX, USA

**Keywords:** Parkinson’s disease, bone mineral density, motor symptoms, levodopa, osteoporosis

## Abstract

**Key points:**

- Significantly lower BMD in PD compared to healthy subjects in both genders.
- Less than 35 mg^2^/dl^2^ of Ca–P product in >80% of PD patients.
- Significant correlations between BMD and severity of PD.
- Lower BMD at H&Y stage III/IV than that at H&Y stage I/II.

**Objectives:**

Although several lines of evidence have suggested that patients with Parkinson’s disease (PD) have a higher risk of osteoporosis and fracture, the association between bone mineral density (BMD) and severity of PD patients is unknown.

**Methods:**

We performed a cross-sectional study of 54 patients with PD and 59 healthy age-matched controls. Multiple clinical scales were used to evaluate the severity of PD, and serum levels of calcium, phosphorus, and homocysteine were measured to determine BMD’s association with PD severity.

**Results:**

BMD in PD patients was significantly lower than that in healthy controls. The BMD scores of the spine, femoral neck (FN), and hip were lower in females than in males in the healthy group. In the PD group, BMD in the hip was significantly lower in females compared to males. There was a negative correlation between daily l-DOPA dosage and BMD in the spine and hip in the PD group, while BMD in the spine, neck, and hip was significantly correlated with severity of PD. Besides, we found that among the lumbar spine (LS), FN, and hip, bone loss in the LS was the most severe in PD patients based on the *T*-scores.

**Conclusion:**

Our findings support the hypothesis that patients with PD have a higher risk of osteoporosis, and that low BMD in the spine, FN, and hip may indirectly reflect the severity of PD. Our findings have prompted us to pay more attention to osteoporosis in the LS in Chinese PD patients.

## Introduction

Parkinson’s disease (PD) is typically characterized by motor symptoms (MS) and non-motor symptoms (NMS). All of the MS and NMS damage the quality of daily life (Bryant et al., 2015). Among the NMS, osteoporosis, a metabolic skeletal disorder characterized by low bone mass and the microarchitectural deterioration of bone tissue in aged subjects, affects a majority of PD patients. Osteoporosis is recognized as a dysfunction in terms of microarchitectural deterioration and low bone mass [*T*-score: <−2.5 standard deviations (SDs)], which increases skeletal fragility (Kanis, 2002; Kanis et al., 2008). It has been well documented that both calcium and phosphorus play key roles in the formation of new bone and in the maintenance of existing bone (Hong et al., 2013). An increase in the ratio of serum calcium concentration and phosphorus concentration (Ca/P) significantly decreases the risk for osteoporosis (Hong et al., 2013). It has been reported that older females are susceptible to osteoporosis due to their decreased levels of estrogen (Tyagi et al., 2012; Cui et al., 2013). On the other hand, numerous studies have indicated that fracture-related morbidity and mortality are higher in older men than in women, and osteoporotic fractures usually occur at approximately 70–75 years old (Gielen et al., 2011). Currently, several epidemiological studies have confirmed that bone mineral density (BMD) is the single best predictor of future fracture (Cooper and Melton Iii, 1992; Allen et al., 2012). Although there were previous studies showing the BMD changes in PD patients, most of these studies were undertook in Western countries and some Asian countries such as Japan and Korea. There is no study yet from Chinese population. Since there are huge differences in ethnicity, food, and life habit between Chinese and Westerners and among Asia populations such as Japanese, it is necessary to determine if there are any specific changes in BMD of Chinese PD patients as compare to that in other countries.

Patients with PD have a higher risk of fallings, osteoporosis, and fracture (Genever et al., 2005; Fink et al., 2008; Abou-Raya et al., 2009). Several lines of evidence suggest that patients with PD fall frequently compared to healthy subjects (Gazibara et al., 2015). The factors that affect the likelihood of falling in PD patients can be divided into two categories: PD-related and PD-unrelated (e.g., age-related falling and fracture) factors. The PD-related factors include poor balance, freezing of gait, poor lower extremity sensation and lower extremity weakness, depression, and the severity of the disease. Homocysteine (Hcy) levels have recently attracted wide clinical attention as a predictor of PD progression. Elevated plasma levels of Hcy have been observed in PD patients (Zhang et al., 2011), and Hcy is known to participate in bone remodeling (Vacek et al., 2013). However, it remains unknown if serum Hcy is correlated with severity of PD.

We undertook this study to determine whether BMD could predict or evaluate the outcomes of motor/non-motor status in patients with PD. The primary goal of this study was to compare the levels of different clinical parameters between PD and healthy subjects. The second aim was to assess whether gender plays an important role in the pathogenesis of bone loss and bone osteoporosis in PD patients. The third purpose of this study was to conduct an exploratory analysis to identify any association between BMD and PD progression, especially with respect to poor motor function and l-DOPA dosage and any association between BMD and Hoehn and Yahr stages (H&Y).

## Materials and Methods

### Patients, Ethics Statement, and Study Design

From July 2011 to September 2015, a total of 54 patients with PD (Table 1) who were admitted to the Department of Neurology of the Third Affiliated Hospital of Sun Yat-sen University, Guangzhou, P. R. China were enrolled in this cross-sectional study. The patients were identified according to the United Kingdom IPD Society Brain Bank (UK-PDSBB) criteria (Hughes et al., 1992). In addition, 59 healthy control subjects matched for age and sex were recruited from the outpatient population as the control group. PD patients were excluded if they had other known causes of osteoporosis, cerebrovascular disease, renal, hepatic or thyroid impairment, or a history of therapy with corticosteroids, estrogen, bisphosphonates, calcitonin, calcium, or vitamin D. The study was approved by the local Ethics Committee of the Third Affiliated Hospital of Sun Yat-sen University and conducted according to the principles outlined in the Declaration of Helsinki of 1975. All of the participants provided written consent for the investigation and for the measurements using the unified Parkinson’s disease rating scale (UPDRS), the Schwab & England activities of daily living scale (S&E), the Webster scale, and the H&Y scale (Hoehn and Yahr, 1967). The demographics and clinical data of the subjects are shown in Table 1.

**Table 1:** **Demographic, motor, and non-motor parameters of PD patients**.

Clinical parameters	PD		
	Mean (±SD)	Min	Max
Gender (*n*)			
Male (%)	29 (53.7)		
Female (%)	25 (46.3)		
Age (years)	65.00 (9.00)	48	80
Disease duration (years)	3.99 (2.88)	1	10
BMI	21.88 (2.95)	16.00	29.82
Daily l-DOPA dosage (mg)	377.54 (129.76)	100	700
Webster	8.01 (3.43)	4	18
S&E (%)	76.68 (13.17)	50	90
H&Y	2.11 (0.816)	1	4
UPDRS II	18.22 (5.40)	6	27
UPDRS III	21.42 (11.89)	11	33
Ca (mg/dl)	2.352 (0.14)	2.1	2.7
P (mg/dl)	1.043 (0.15)	0.77	1.39
Ca*P (mg^2^/dl^2^)	31.32 (4.54)	22.84	43.52
Ca–P product <35 (mg^2^/dl^2^) *n* (%)	45 (83.3)		
Ca–P product ≥35 (mg^2^/dl^2^) *n* (%)	9 (16.7)		
Hcy (μmol/l)	13.49 (4.79)	5.44	35.02

BMI, body mass index; Ca, calcium; Ca*P, serum Ca concentration and phosphorus concentration product; Hcy, homocysteine; H&Y, the modified Hoehn and Yahr staging scale; l-DOPA, levodopa; P, phosphorus; PD, Parkinson’s disease; UPDRS, unified Parkinson’s disease rating scale.

### Blood Sampling Measurement

Venous blood samples were collected in the early morning under fasting condition in order to obtain serum Ca, P, and plasma Hcy measurements in the study. Concentrations of Ca and P were ascertained according to certified methods of the Institute of Clinical Chemistry and Laboratory Medicine, the Third Affiliated Hospital of Sun Yat-Sen University. For Hcy measurement, the blood samples were centrifuged at 3,000 rpm for 10 min and stored at −70° C until the laboratory evaluation. The plasma levels of Hcy were determined using a solid phase competitive chemiluminescent enzyme immunoassay (Rodriguez-Oroz et al., 2009; Zhang et al., 2011). All of blood measurements were replicated thrice.

### Bone Mineral Density Measurement

**Figure 1:**
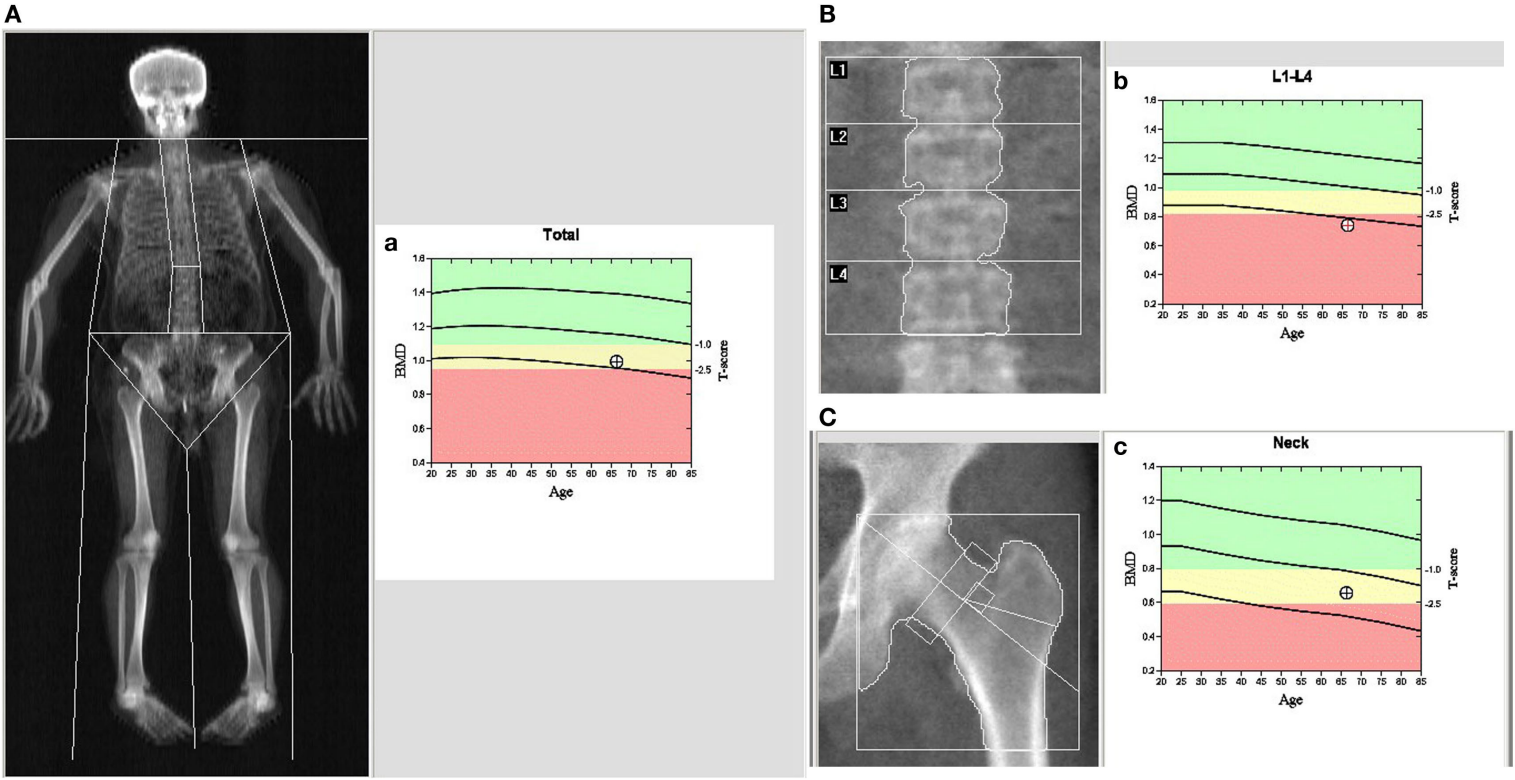
**Dual-energy X-ray absorptiometry (DXA) imaging of BMD in a male PD patient**: **(A)** the whole body, **(B)** spine, and **(C)** left hip. **(a–c)** The patients’ BMD vs. a male Chinese control of the whole body **(a)**, spine **(b)**, and left hip **(c)**. In **(a–c)**, the first cure line stands for baseline in the different ages; the second cure line stands for the boundary between normal BMD and osteopenia; the third cure line stands for the boundary between osteopenia and osteoporosis, and the round dot stands for the patients’ BMD.

Bone mineral density (g/cm^2^) of the lumbar spine (LS), femoral neck (FN), and hip were measured using a dual-energy X-ray absorptiometry (DXA) machine (model, Discovery A) according to the manufacturer’s guidelines (Kanis, 2002; Wood and Walker, 2005; Kanis et al., 2008). Before measurement, routine quality control was performed to make sure our device was accurate and all the subjects underwent the measurement after bladder emptying. The least significant change (LSC) at our department was calculated to be 0.041 (LS), 0.042 (hip), and 0.035 g/cm^2^ (FN), within the limits of International Society for Clinical Densitometry (Xie et al., 2013). *Z*- and *T*-scores were automatically calculated by the software in the work station of DXA, where the *Z*-score is the SD of the individual’s BMD compared to the mean BMD score of a similar sex- and age-matched population, and the *T*-score is the SD of the individual’s BMD compared with the mean BMD score of a young, healthy population. For menopausal women and men over 50 years old, BMD was categorized as normal, osteopenia (low bone density) or osteoporosis, as defined by the World Health Organization (WHO) (Gielen et al., 2011). A typical BMD image of the PD patients is shown in Figure 1.

### Statistical Analysis

All continuous variables, including age, disease duration, body mass index (BMI), Webster, S&E, UPDRS II, and UPDRS III scores, H&Y stage, and Ca, P, Ca*P, and Hcy levels, were presented as the mean ± SD, and all categorical variables, including gender, Ca–P product <35 (mg^2^/dl^2^), and Ca–P product ≥35 (mg^2^/dl^2^) were presented as numbers and percentages. The statistical significance of differences between the groups was assessed using Student’s *t*-test. And gender difference in affecting BMD in PD was assessed employing two-way ANOVA. Spearman’s rank correlation coefficient (*r*_s_) was used to evaluate the correlations of the different clinical parameters. A *p*-value <0.05 was deemed statistically significant, and SPSS 13.0 software (Chicago, IL, USA) was used for the statistical analyses.

## Results

### Patient Characteristics

The cross-sectional study consisted of 54 PD patients [29 males (53.7%) and 25 females (46.3%)] and 59 healthy subjects [34 males (57.6%) and 25 females (42.4%)] (Tables 1 and 2). Detailed information was shown in Tables 2–4.

**Table 2:** **Comparison of bone mineral density between PD patients and healthy subjects**.

	PD	Control	p
		
	Mean (SD)	Mean (SD)	
Age	65.00 (9.00)	65.85 (9.46)	0.632
Height	161.58 (6.73)	161.01 (8.09)	0.725
Weight	57.10 (8.01)	62.41 (12.01)	0.021
BMI	21.90 (2.90)	23.99 (3.51)	0.004**
LS (g/cm^2^)	0.80 (0.11)	0.93 (0.15)	0.000***
LS T	−2.44 (1.06)	−1.17 (1.29)	0.000***
LS Z	−1.25 (1.08)	0.27(1.21)	0.000***
FN (g/cm^2^)	0.67 (0.12)	0.74 (0.11)	0.002**
FN T	−1.78 (0.95)	−1.12 (0.82)	0.000***
FN Z	−0.51 (0.86)	0.26 (0.74)	0.000***
Hip (g/cm^2^)	0.83(0.11)	0.90 (0.13)	0.004**
Hip T	−1.19 (0.76)	−0.57 (0.84)	0.000***
Hip Z	−0.29 (0.71)	0.48 (0.78)	0.000***

*p < 0.05, **p < 0.01, ***p < 0.001 with Student’s test.

### Comparisons of Bone Mineral Density Between the PD Patients and the Normal Subjects

**Figure 2:**
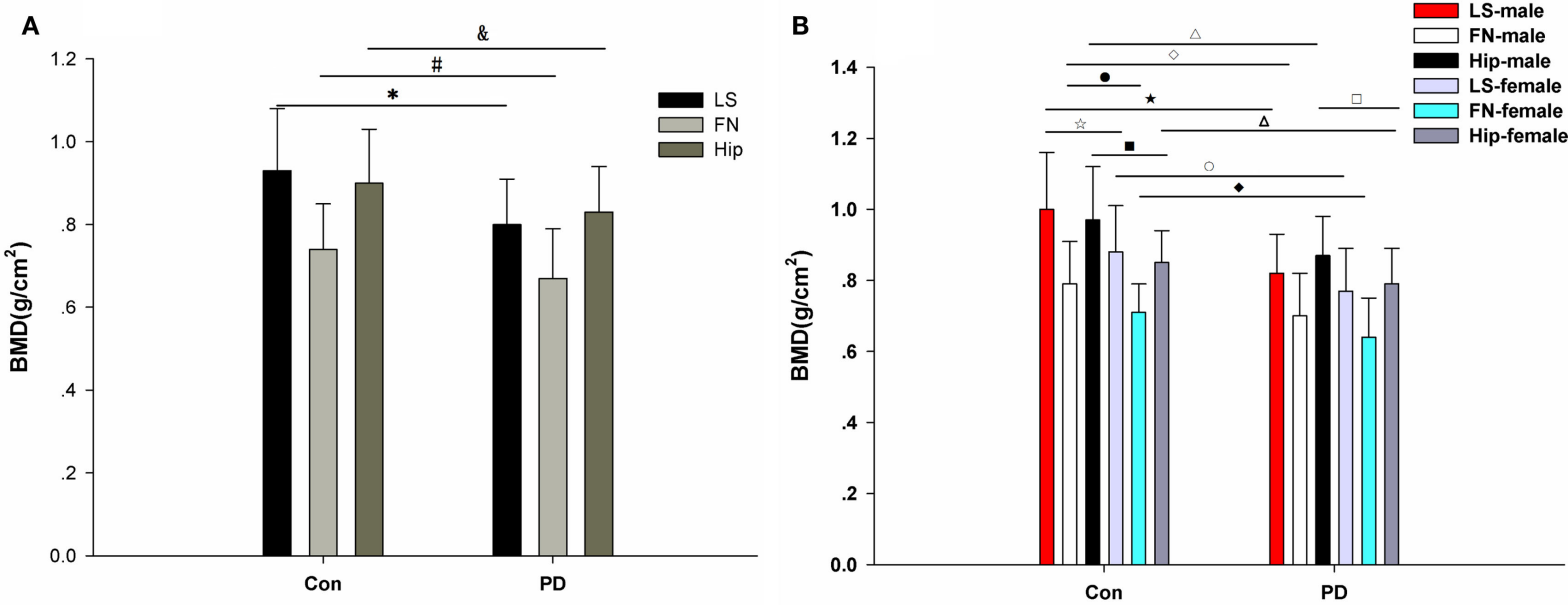
**BMD in PD patients and healthy controls**. **(A)** Comparison of BMD in the lumbar spine (LS), femoral neck (FN), and hip between the matched control group and the PD group. There are significant differences in LS, FN, and hip BMD between the PD patients and the controls. *LS: PD vs. control: 0.80 ± 0.11 vs. 0.93 ± 0.15, ***p < 0.001; #FN: PD vs. control: 0.67 ± 0.12 vs. 0.74 ± 0.11, **p = 0.002; and hip: PD vs. control: 0.83 ± 0.11 vs. 0.90 ± 0.13, **p = 0.004. **(B)** Comparison of BMD in the lumbar spine (LS), femoral neck (FN), and hip between the matched control group and the PD group, which were divided into gender-specific groups. There are significant differences in LS BMD between the control males and the control females (□: 1.00 ± 0.16 vs. 0.88 ± 0.13, **p = 0.005), the PD males and the control males (□: 0.82 ± 0.11 vs. 1.00 ± 0.16, ***p < 0.001), and the PD females and the control females (○: 0.77 ± 0.12 vs.0.88 ± 0.13, **p = 0.002). There are significant differences in FN BMD between the control males and the control females (●: 0.79 ± 0.12 vs. 0.71 ± 0.08, **p = 0.003), the PD males and the control males (□: 0.70 ± 0.12 vs. 0.79 ± 0.12, **p = 0.008), and the PD females and control females (□: 0.64 ± 0.11 vs. 0.71 ± 0.08, *p = 0.012). There are significant differences in hip BMD between the PD males and PD females (□: 0.87 ± 0.11 vs. 0.79 ± 0.10, **p = 0.007), the control males and the control females (□: 0.97 ± 0.15 vs. 0.85 ± 0.09, **p = 0.001), the PD males and the control males (Δ: 0.87 ± 0.11 vs. 0.97 ± 0.15, **p = 0.007), and the PD females and the control females (∆: 0.79 ± 0.10 vs. 0.85 ± 0.09, **p = 0.009).

No significant difference in age, height, or weight was observed between the PD patients and the healthy controls (Table 2) in this study. However, a significant difference in BMI was observed between the PD patients and the healthy controls (**p = 0.004; Table 2). Moreover, the LS BMD in the PD patients was remarkably lower than that in the controls (***p < 0.001, Table 2). Accordingly, the *T*-scores and *Z*-scores for the LS BMD were decreased in the PD patients compared with those in the healthy subjects (Table 2). Furthermore, similar differences were found in the FN and hip between the PD patients and the matched controls. The FN BMD in the PD patients was 0.67 ± 0.12 g/cm^2^, while it was 0.74 ± 0.11 g/cm^2^ in the control group. The hip BMD was 0.83 ± 0.11 vs. 0.90 ± 0.13 g/cm^2^, *p = 0.004 (Table 2; Figure 2A). The results of the comparisons of the *T*-scores and *Z*-scores for the FN were as follows: *T*-score, −1.78 ± 0.95 vs. −1.12 ± 0.82, ***p < 0.001; *Z*-score, −0.51 ± 0.86 vs. 0.26 ± 0.74, ***p < 0.001; and for the hip were: *T*-score, −1.19 ± 0.76 vs. −0.57 ± 0.84, ***p < 0.001; *Z*-score, −0.29 ± 0.71 vs. 0.48 ± 0.78, ***p < 0.001 (Table 2). Moreover, among the LS, FN, and hip, bone loss in the LS was the most serious in PD patients based on the *T*-scores.

### Gender Differences in Affecting BMD in PD

Because gender is one of the factors that influences BMD, we further analyzed BMD in the different genders. Our results showed that significant differences were observed in the BMD of the LS, FN, and hip between healthy males and healthy females (Tables 3 and 4). Significant differences were observed for BMI, height, LS *Z*-score, and hip BMD when comparing male PD patients with female PD patients (Tables 3 and 4). Significant differences were found between the male PD patients and the male healthy controls in terms of BMI (*p = 0.024), weight (*p = 0.034), LS BMD (***p < 0.001), LS *T*-score (***p < 0.001), LS *Z*-score (***p < 0.001), FN BMD (**p = 0.008), FN *T*-score (*p = 0.012), FN *Z*-score (**p = 0.001), hip BMD (**p = 0.007), hip *T*-score (**p = 0.005), and hip *Z*-score (**p = 0.002) (Table 4). In the female PD and healthy cohorts, there were also significant differences in the LS BMD (**p = 0.002), LS *T*-score (**p = 0.001), LS *Z*-score (***p < 0.001), FN BMD (**p = 0.012), FN *T*-score (**p = 0.009), FN *Z*-score (**p = 0.002), and hip BMD (**p = 0.009), LS *T*-score (**p = 0.005), *Z*-score (**p = 0.001) (Figure 2B).

**Table 3:** **Demographic data and bone mineral density of PD patients: gender differences**.

Subjects	PD male (SD)	PD female (SD)	Con male (SD)	Con female (SD)
Age	66.19 (9.29)	63.62 (8.76)	66.71 (9.18)	65.36 (9.71)
BMI	21.65 (2.52)	22.11 (3.38)	23.96 (3.43)	24.00 (3.60)
Height	165.61 (3.91)	157.56 (6.61)	167.84 (7.65)	157.03 (5.20)
Weight	59.44 (7.74)	54.75 (7.77)	68.16 (15.16)	59.05 (8.24)
LS	0.82 (0.11)	0.77 (0.12)	1.00 (0.16)	0.88 (0.13)
LS *T*-score	−2.43 (1.08)	−2.46 (1.05)	−0.74 (1.38)	−1.43 (1.25)
LS *Z*-score	−1.66 (0.98)	−0.77 (1.02)	0.16 (1.35)	0.34 (1.14)
FN	0.70 (0.12)	0.64 (0.11)	0.79 (0.12)	0.71 (0.08)
FN *T*-score	−1.69 (0.91)	−1.90 (1.00)	−1.0 (0.85)	−1.26 (0.80)
FN *Z*-score	−0.59 (0.80)	−0.42 (0.94)	0.21 (0.79)	0.30 (0.71)
Hip	0.87 (0.11)	0.79 (0.10)	0.97 (0.15)	0.85 (0.09)
Hip *T*-score	−1.10 (0.70)	−1.29 (0.84)	−0.40 (0.95)	−0.68 (0.76)
Hip *Z*-score	−0.46 (0.68)	−0.10(0.72)	0.31 (0.92)	0.58(0.69)

**Table 4:** **Gender differences in bone mineral density in PD patients vs. healthy subjects**.

*p*	PD: male vs. female	Con: male vs. female	Male: PD vs. Con	Female: PD vs. Con
Age	0.318	0.607	0.844	0.486
BMI	0.624	0.964	0.024*	0.069
Height	0.000***	0.000***	0.271	0.750
Weight	0.078	0.005**	0.034*	0.071
LS	0.192	0.005**	0.000**	0.002**
LS *T*-score	0.918	0.053	0.000**	0.001**
LS *Z*-score	0.003**	0.599	0.000**	0.000***
FN	0.094	0.003**	0.008**	0.012*
FN *T*-score	0.430	0.261	0.012*	0.009**
FN *Z*-score	0.491	0.670	0.001**	0.002**
Hip	0.007**	0.001**	0.007**	0.009**
Hip *T*-score	0.382	0.229	0.005**	0.005**
Hip *Z*-score	0.069	0.202	0.002**	0.001**

Two-way ANOVA, *p < 0.05, **p < 0.01, ***p < 0.001 with two-way ANOVA.

### Correlations Between BMD and Age, l-DOPA Dose, S&E, Webster, UPDRS II, and UPDRS III Scores as Well as H&Y Stage

There were significant negative correlations between age and LS BMD (*r*_s_ = −0.195, *p = 0.044), *T*-score (*r*_s_ = −0.202, *p = 0.037), FN BMD (*r*_s_ = −0.226, *p = 0.019), FN *T*-score (*r*_s_ = −0.249, *p = 0.010), hip BMD (*r*_s_ = −0.153, *p = 0.029), and hip *T*-score (*r*_s_ = −0.217, *p = 0.025) (Table 5). This means that in the PD patients, BMD decreased as age increased. Significant negative correlations were also found between daily l-DOPA dosage and LS BMD (*r*_s_ = −0.443, *p = 0.019), LS *T*-score (*r*_s_ = −0.388, *p = 0.033), hip BMD (*r*_s_ = −0.422, *p = 0.023), and hip *T*-score (*r*_s_ = 0.372, *p = 0.038) (Table 5). We observed significant negative correlations between BMD (*T*-score and *Z*-score) and Webster, UPDRS II, and UPDRS III scores as well as H&Y stage, and a positive correlation between BMD and S&E score, with no significant correlations between LS BMD and UPDRS II score (*p* = 0.090). Therefore, our data show that disease severity was increased in the patients who experienced greater bone loss.

**Table 5:** **Correlations between BMD, Hcy and age, l-DOPA, S&E, Webster, UPDRS II, UPDRS III, H&Y in PD patients**.

	age		BMI		Hcy		l-DOPA		S&E		Webster		UPDRS II		UPDRS III		H&Y	
	*r*	*p*	*R*	*p*	*r*	*p*	*r*	*p*	*r*	*p*	*r*	*p*	*r*	*p*	*r*	*p*	*r*	*p*
LS	−0.195	0.044*	0.247	0.017*	−0.026	0.780	−0.443	0.019*	0.419	0.008**	−0.519	0.002**	−0.281	0.090	−0.423	0.007**	−0.364	0.036*
T	−0.202	0.037*	0.260	0.012	−0.066	0.675	−0.388	0.033*	0.492	0.009**	−0.575	0.000***	−0.404	0.020*	−0.488	0.003**	−0.378	0.021**
Z	0.072	0.462	0.347	0.001**	−0.084	0.773	−0.153	0.561	0.488	0.004**	−0.455	0.003**	−0.366	0.027*	−0.472	0.002**	−0.455	0.002**
FN	−0.226	0.019*	0.174	0.095	−0.111	0.509	−0.361	0.098	0.488	0.003**	−0.598	0.000***	−0.356	0.021*	−0.466	0.003**	−0.401	0.016*
T	−0.249	0.010*	0.182	0.081	−0.126	0.396	−0.295	0.122	0.565	0.000***	−0.625	0.000***	−0.428	0.009**	−0.509	0.001**	−0.443	0.004**
Z	0.112	0.255	0.272	0.009**	−0.541	0.372	−0.137	0.478	0.571	0.000***	−0.644	0.000**	−0.461	0.003**	−0.531	0.002**	−0.509	0.002**
Hip	−0.153	0.029*	0.169	0.106	−0.066	0.736	−0.422	0.023*	0.518	0.002**	−0.371	0.016*	−0.365	0.022*	−0.539	0.001**	−0.0408	0.015*
T	−0.217	0.025*	0.200	0.055	−0.123	0.569	−0.372	0.038*	0.618	0.000***	−0.652	0.000***	−0.435	0.005**	−0.616	0.000***	−0.511	0.002**
Z	0.158	0.105	0.297	0.004**	−0.131	0.433	−0.124	0.588	0.627	0.000***	−0.596	0.000***	−0.497	0.002**	−0.612	0.000***	−0.496	0.000***
Hcy	0.128	0.453	−0.239	0.310	1	−	0.600	0.000**	−0.336	0.059	0.491	0.011*	0.410	0.011*	0.474	0.007**	0.286	0.073

*p < 0.05 level (two-tailed); **p < 0.01; ***p < 0.001.

### Different Changes in Bone Mineral Density in the PD Patients at Different H&Y Stages

To explore the relationship between BMD and the severity of MS in different stages of PD, the patients were categorized into two groups according to the H&Y scale: group one for H&Y I and II and group two for H&Y III and IV. A significant difference was observed between the two groups in BMD, including LS BMD, **p = 0.005, *T*-score of spine, **p = 0.003; *Z*-score of spine, ** *p* = 0.002; FN BMD, *p = 0.027; *T*-score of FN, *p = 0.015; *Z*-score of FN, **p = 0.007, hip BMD, **p = 0.004; *T*-score of hip, ***p < 0.001; and *Z*-score of hip, **p = 0.001 (Table 6).

**Table 6:** **Comparisons of bone mineral density in PD patients at different stages of H&Y**.

	H&Y I and II	H&Y III and IV	*P*
Age	64.31 (6.63)	65.01 (9.26)	0.152
Duration (years)	3.25 (2.55)	4.12 (3.64)	0.232
LS	0.845 (0.11)	0.75 (0.10)	0.005**
LS T	−1.94 (0.88)	−3.01 (0.79)	0.003**
LS Z	−0.74 (0.87)	−1.93 (0.98)	0.002**
FN	0.69 (0.10)	0.61 (0.10)	0.027*
FN T	−1.66 (0.70)	−2.35 (0.78)	0.015*
FN Z	−0.36 (0.59)	−1.13 (0.67)	0.007**
Hip	0.86 (0.08)	0.77 (0.12)	0.004**
T	−0.93 (0.46)	−1.64 (0.61)	0.000***
Z	−0.077 (0.56)	−0.507 (0.55)	0.001**

*p < 0.05; **p < 0.01; ***p < 0.001 with Student’s test.

## Discussion

Previous studies have indicated BMD changes in PD patients (Genever et al., 2005; Di Monaco et al., 2006; Abou-Raya et al., 2009; Song et al., 2009; Lam et al., 2010; Sato et al., 2011; Daniel et al., 2012; Zhao et al., 2013). Some studies investigated the risk rate of PD in terms of osteopenia/osteoporosis/fracture (Sato et al., 2001; Fink et al., 2005, 2008; Latourelle et al., 2010; Lee et al., 2010). Others focused on the factors predisposing PD patients to low BMD and factors improving osteoporosis (Lam et al., 2010): low percentage of body fat (Lam et al., 2010), low vitamin D, increased bone alkaline phosphatase (BALP) (Sato et al., 2005a,b; Abou-Raya et al., 2009), and sunlight exposure (Sato et al., 1999, 2011), respectively, the relationship between the PD disease duration and osteopenia/osteoporosis(Daniel et al., 2012). Considering that the factors influencing BMD vary from different ethnicity to different country regions (Kao et al., 1994; Torsney et al., 2014), it is very important to investigate the bone conditions in the mainland of China. From the PubMed searching, we found no BMD study from the mainland of China. This study first investigated the bone conditions of Chinese PD patients from Mainland China. It combined BMD, Ca–P product, and Hcy levels, S&E, Webster, UPDRS II, and UPDRS III scores, and H&Y stages to assess the changes in BMD and its potential roles in the progression and severity of Chinese PD patients. We used the S&E and Webster based on the following reasons: (1) Webster consists of 10 indexes for more details of PD symptoms and gave the direction that we should pay more attention to the MS such as the gait parameter and standing balance in the patients; (2) a combination of mutiple scales, including S&E, Webster, H&Y and the UPDRS, allowed us to explore the relationship between the severity of PD and osteoporosis and to obtain more reliable results. Therefore, our data gained in this way may provide us with more persuasive and solid conclusion. From the Table 5, we note that the higher dose of l-DOPA usage and PD severity as indicated by S&E, Webster, UPDRS II, UPDRS III, and H&Y scores are negatively correlated to the BMD scores, strongly implying that as the disease develops, osteogenesis will be seriously prevented and cause osteopenia. It indicates that the greater the severity of PD and the higher the dose of l-DOPA, the more serious the osteopenia with a decreased BMD, suggesting that the relationship of l-DOPA dosage with BMD changes may give insights into the safety of PD therapy. Ca and P are the main bone forming minerals. They affect the levels of PTH and are essential in bone formation and development. Previous studies suggested that ionized calcium (Sato et al., 2005a,b; Abou-Raya et al., 2009), 25-hydroxyvitamin D (25-OHDA), and 1,25-dihydroxyvitamin D (1,25-[OH](2)D) (Sato et al., 2005a,b) have a close relationship with osteoporosis in PD patients. As Ca and P serum levels may be altered by some metabolism materials and can interact with each other, here we employed the Ca–P product to evaluate BMD in PD patients. When the Ca–P product is <35 mg^2^/dl^2^, the conditions are not appropriate for calcification and may cause the bone salt to dissolve, preventing osteogenesis. As shown in Table 1, although the serum Ca and P levels were within the normal range, the Ca–P product in most of the PD patients (83.3%, Table 1) was <35 mg^2^/dl^2^, indicating that the majority of the PD patients’ bones were in a stage in which bone had a tendency to dissolve, with little osteogenesis.

Female steroid hormones may play an important role in osteoporosis, which affects approximately 30% of females and 8% of males who are over 50 years old (Kwan, 2015). Osteoporosis is not only associated with age but also with gender – females have a higher risk of osteoporosis. Individuals with PD also have a higher risk of osteoporosis, and gender is an independent factor in PD patients as well. In the current study, we aimed to explore the relationship between BMD and the symptom severity of PD patients according to gender.

As the optimal available method to predict future fracture, BMD is inversely correlated with fracture. There were major controversies in some previous reviews and meta-analyses, mainly due to the difference in literature-searching criteria. For example, the meta-analysis by Torsney et al. used the following terms as the search strategy: “Osteoporosis and PD,” “Osteopenia and PD,” “Fracture and PD,” and “Bone health and PD” and showed that female patients were at greater risk of osteoporosis (Torsney et al., 2014). However, a recent meta-analysis by Zhao et al. used different search terms such as “Parkinson’s disease and osteoporosis” or “Parkinson’s disease and bone mineral density” and showed the opposite results, indicating that PD male patients to be at a higher risk for osteoporosis than female patients (Zhao et al., 2013). Another main reason for this discrepancy may result from that fact that Zhao et al. chose different criteria, including the use of abstracts, non-English language articles and an article in which osteoporosis preceded the diagnosis of PD. These differences might account for the controversial evidences between the two meta-analyses (Zhao et al., 2013; Torsney et al., 2014).

In the current study, among the LS, FN, and hip, the LS was the most fragile, as its *T*-score was the lowest (Table 2), which explains why patients with PD have more than twice the risk for fractures than an age-matched cohort (Melton et al., 2006). In this study, we found that among the LS, FN, and hip, bone loss in the LS was the most severe in PD patients based on the *T*-scores. This finding is different from those of previous studies. Some studies from Western countries such as UK and Turkey show that the increased risks for osteoporosis or higher fracture risks in patients with PD normally occur in hip and proximal femur, respectively (Kamanli et al., 2008; Lyell et al., 2015). Bone loss was more severe in FN than lumber spine in PD patients from Egypt (Abou-Raya et al., 2009) and Korea (Song et al., 2009). In Japan, some studies showed that hip has a higher fracture risk in PD patients (Sato et al., 1999, 2001). Our findings have prompted us to pay more attention to osteoporosis in the LS in Chinese PD patients, unlike in those of other Asian countries or western countries.

Moreover, because the BMD of the female PD patients was lower than that of the male PD patients (Tables 3 and 4), we propose that being female was an independent risk factor for decreased bone density in PD patients. Several lines of evidence have shown that osteoporosis varies between men and women due to the uncoupling of bone remodeling as a result of sex hormone deficiencies, menopause, vitamin D deficiency, reduced synthesis of D hormone, and the lack of receptors or reduced receptor affinity for D hormone in target organs (Liao et al., 2014).

We may see that BMD values were significantly different between males and females in normal people from Table 4. However, when comparing PD males to females, BMD values in the LS and FN showed no significant difference. When comparing BMD between PD patients and the controls by genders, there were significant differences in all of the BMD indexes between the PD males and the control males, but not all of them were significantly different between the female PD and female healthy subjects (Table 4). It seems that the BMD of both LS and FN in PD male and female patients becomes lower in comparison to healthy subjects, but the decrease is more pronounced in male PD patients, subsequently leading to the disappearance of differences between males and females with PD. We propose that BMD is more affected in PD males than in PD females, and the hip is probably less affected by osteoporosis in female PD patients compared to male PD patients. According to our findings (Table 4; Figure 2B), we hypothesize that the effect of genders on bone development may play an important role in PD patients.

Significant Spearman’s correlations were observed between BMD and the severity of PD, strongly indicating that the grade of osteoporosis may reflect the progression of PD. We therefore propose the following reasons for osteoporosis: (1) tremor, bradykinesia, and rigidity obviously decrease outdoor activities, including weight-bearing exercise, which may increase bone deposition and decrease bone re-absorption (Rison and Richardson, 2011); and (2) some NMS, such as depression and dementia, cause patients to be unwilling to participate in daily outdoor activities and to get less sunlight exposure (Rison and Richardson, 2011). This hypothesis is indirectly reflected in our results (Table 5). Furthermore, to obtain deeper insight into whether BMD levels vary according to the different stages of PD, we divided those PD subjects into two stages, H&Y I/II and III/IV, indicating the early and later stages, respectively, and compared BMD scores between these two stages. Our results (Table 6) further demonstrated that the osteoporosis in PD becomes worse in accordance with disease severity and progression.

Giving l-DOPA as a treatment can improve mobility and postural stability in PD; however, as the l-DOPA dosage increases, side effect such as dyskinesia may occur due to the high dose of l-DOPA. This could cause the patient to fall down more easily and aggravate the risk of fracture. According to the close relationship between PD and lower BMD, effective preventive strategies that enable PD patients to increase bone density, prevent excessive bone loss, and decrease the risk of falls are urgently needed. Treatment with bisphosphonates, vitamin D, and calcium can increase BMD and reduce fractures in PD patients. The l-DOPA-related adverse effects that are closely associated with falls and fracture mainly include two aspects: (1) motor fluctuations and dyskinesias (Jankovic, 2005); and (2) hyperhomocysteinemia, which leads to an increase in the destruction of bone tissue, a decrease in BMD, and the development of osteopenia and osteoporosis (Rico et al., 1979; Bezsmertnyi Iu, 2013). It seems that bone loss in PD is multifactorial, including immobility, decreased muscle strength, low body weight, and vitamin D deficiency.

Several limitations of our study should be noted: (1) a small number of participants were recruited and it is necessary to conduct large population studies in the future; (2) there were no patients with PD at the late stage of the disease, as indicated by PD patients at H&Y from I to IV; (3) the daily intake of vitamin D and sunlight exposure were not considered in this study, and this study does not involve more assessment markers such as ionized Ca about bone formation and bone metabolism; and (4) in order to validate and complete the questionnaire, we only chose PD subjects with sufficient cognitive ability, significantly narrowing the population of the study. The limitations described above regarding the population chosen may have resulted in a bias for Ca, P, Hcy, and BMD levels in the PD. Thus, it is necessary to conduct larger population studies. However, this study still provides first and comprehensive data regarding the bone conditions of Chinese PD patients from Mainland China.

In summary, the current study supports the notion that PD patients have a higher risk for osteoporosis. In the healthy controls, female gender was an independent risk factor for osteoporosis. In addition, age could worsen bone loss and osteoporosis. Thus, our findings may provide a valuable hint for therapeutic strategy for treating osteoporosis in PD patients. Based on our findings, the examination of BMD using DXA is a valid way to detect osteoporosis in PD. Because of the prevalence of bone problems in PD, the therapeutic interventions should be initiated to improve the quality of life in PD.

## Author Contributions

Conceived and designed the experiments: HG, XW, CL, RW, and QW. Performed the experiments: HG, RW, LX, and QW. Analyzed the data: HG, RW, JX, and QW. Contributed reagents/materials/analysis tools: XP, ZL, and ZL. Wrote the paper: HG, YX, and QW.

## Conflict of Interest Statement

The authors declare that the research was conducted in the absence of any commercial or financial relationships that could be construed as a potential conflict of interest.

## Abbreviations

BMD, bone mineral density; BMI, body mass index; Ca, calcium; Ca–P product, serum calcium concentration and phosphorus concentration product; DXA, dual-energy X-ray absorptiometry; FN, femoral neck; Hcy, homocysteine; H&Y, Hoehn and Yahr staging scale; l-DOPA, levodopa; LS, lumbar spine; LSC, least significant change; MMPs, matrix metalloproteinases; MS, motor symptoms; NMS, non-motor symptoms; P, phosphorus; PD, Parkinson’s disease; PTH, parathyroid hormone; RBD, rapid eye movement sleep; rs, the Spearman’s rank correlation coefficient; SD, standard deviation; S&E, the Schwab & England activities of daily living scale; UK-PDSBB, the United Kingdom IPD Society Brain Bank; UPDRS, unified Parkinson’s disease rating scale; 25-OHDA, 25-hydroxyvitamin D; 1,25-[OH](2)D, 1,25-dihydroxyvitamin D.
